# Editorial: Teamwork in human-machine teaming

**DOI:** 10.3389/fpsyg.2022.999000

**Published:** 2022-08-24

**Authors:** Gregory Funke, Joseph B. Lyons, Eric T. Greenlee, Michael T. Tolston, Gerald Matthews

**Affiliations:** ^1^Air Force Research Laboratory, Wright-Patterson Air Force Base, Dayton, OH, United States; ^2^Department of Psychological Sciences, Texas Tech University, Lubbock, TX, United States; ^3^Department of Psychology, George Mason University, Fairfax, VA, United States

**Keywords:** teamwork, human-machine teaming, human-human teaming, team processes, team communication, team trust

Continued development in the fields of artificial intelligence, robotics, and virtual reality means that true human-machine teaming is an impending possibility. Inclusion of these machine agent teammates is expected to provide substantive benefits, including personnel augmentation, access to sophisticated computational abilities, and decision support, among other possibilities (Grigsby, 2018). Here, we use the term “agent” in the same sense as Chen and Barnes (2014), to refer to intelligent systems (with or without physical embodiment) that possess autonomy: the ability to observe and act upon the environment and direct their activity toward achieving certain goals. Introduction of these teammates will also have a profound effect on the dynamics of teamwork (Walliser et al., 2019).

Effective teamwork is a necessary antecedent to team success in human-human teams (Salas et al., 2008). Factors such as leadership, conflict resolution, adaptability, and backup behavior, among many others, have been identified as critical aspects of teamwork supporting team outcomes (e.g., Salas et al., 2005). However, assessing teamwork in human-human teams is often difficult because it comprises behaviors (e.g., communication, coordination), processes (e.g., cooperation, performance monitoring), and emergent states (e.g., trust, shared mental models) that unfold over micro- and macro-temporal scales. At present, there is good reason to believe that the same factors that govern human-human teamwork will also influence human-machine teamwork. Yet, the key to the previous statement is “*at the present,”* because the research necessary to establish and qualify those relationships is still nascent.

Machine agents of the future have been envisioned as sophisticated teammates, able to contribute to team planning and strategy, and capable of executing complex tasks with minimal human oversight (e.g., Zacharias, 2019). To achieve this vision, machine agent teammates must be designed with the understanding that team effectiveness and performance are not determined solely by the simple aggregate of separate member abilities and inputs. Instead, team effectiveness depends upon the successful integration and coordination of individual efforts through team processes and teamwork. These team process elements are reflected in contemporary definitions of human-autonomy teammates, which emphasize the ability to assume a role as a team player and to communicate on behalf of that role for both essential task work as well as teamwork (McNeese et al., 2018). As such, the success of future human-machine teams will depend, in part, on machine agents that have been designed to successfully facilitate and participate in teamwork with human teammates. The articles featured in this special issue aim to meet this goal.

## Research topic insights

This special issue comprises eight manuscripts that span a variety of topics pertinent to human-machine teaming. First are two review papers that identify and present potential solutions for critical gaps that could hinder human-machine teaming. Lyons et al. review the differences between automation and autonomy and examine human-human teams to elucidate core teamwork factors (intent inference, shared mental models, and team-oriented communications) relevant to human-machine teaming. In their review, Stowers et al. present techniques derived from computer science that would harness artificial intelligence to enhance machine agents, thereby enabling superior contribution to critical team competencies and effective human machine teaming.

Next, several authors in the special issue examined the effects of specific features and capabilities of machine agents on human teammates' perceptions and team performance. Bibyk et al. explore how common ground and norms in communication may develop idiosyncratically across teams, but may also be strongly influenced by the instructions and examples provided to the team, and how beliefs about agent capabilities may detrimentally constrain team communication. Sebo et al. examine how an agent's verbal support of outgroup team members encouraged them to participate more in a group task, and increased those outgroup members' feelings of psychological safety and inclusion in the group, but also reduced verbal backchannels from ingroup to outgroup members. Fraune investigated the effects of agent anthropomorphism and group membership on punishment assignments and beliefs about in- and out-group members, finding that people treat ingroup members, including agents, better than outgroup members. Haring et al. adapted Milgram's (1963) paradigm to examine how anthropomorphism and physical embodiment influence compliance with a robot coach's instructions to continue working on a boring task; as expected, they found that compliance was greatest to a human coach, and while participants did comply with a robot coach, their compliance was only weakly affected by manipulations of anthropomorphism and embodiment.

Finally, this volume includes papers that focus on trust in human-machine teams. Kohn et al. review and categorize self-report, behavioral, and physiological methods for assessing trust in automation, including recommendations for improving measurement of trust in automation. Lin et al. explore trust in a robot teammate while considering how individual differences (tendency to view robots as either tools or teammates) and the type of judgment being made by the robot (physics-based or psychology-based) impacted trust.

## The road ahead

Considering that limited forms of human-machine partnerships have already begun (e.g., with semi-autonomous vehicles), further research exploring the factors that contribute to success in human-machine teaming is urgently needed. The field will also grow and change as the technology enabling machine intelligence advances, first as semi-autonomous teammates, and later as true artificial intelligence becomes possible.

The articles included in this special issue all make meaningful contributions to this important topic area, but much is also still left to do. As indicated above, a full examination of teamwork must address it as behaviors, processes, and emergent states—across multiple time scales, suggesting that progress in this area will require dedicated researchers, clever hypotheses, and insightful new theories. We are happy to have interacted with some of those researchers in developing this special issue and we feel that this set of papers will advance the state of knowledge in this burgeoning domain.

## Author contributions

All authors listed have made a substantial, direct, and intellectual contribution to the work and approved it for publication.

## Conflict of interest

The authors declare that the research was conducted in the absence of any commercial or financial relationships that could be construed as a potential conflict of interest.

## Publisher's note

All claims expressed in this article are solely those of the authors and do not necessarily represent those of their affiliated organizations, or those of the publisher, the editors and the reviewers. Any product that may be evaluated in this article, or claim that may be made by its manufacturer, is not guaranteed or endorsed by the publisher.
